# Supplementary material for: Content-rich biological network constructed by mining PubMed abstracts
Source: BMC Bioinformatics. 2004 Oct 8;5:147. doi: 10.1186/1471-2105-5-147 (PMC528731; doi:10.1186/1471-2105-5-147)
Supplement: Additional File 2 — The original results of the above study (non-essential files are deleted to keep the file size under the limit set by BMC bioinformatics). [file 1471-2105-5-147-S2.bz2 › chilibotAdditionalFile2/dip50/152ID8479754E547/html/CCND1_CDK2.html]

 


 **CCND1** and **CDK2** 
  
Found 643 abstracts in PubMed, retrieved 50.  
 

 What does Google say? 
 PDF only 
| .edu only 

---

**Interactive relationship** (e.g. stimulation, inhibition, etc)

**Inhibitory relationship**- Analyses in SK Hep 1 cells revealed a deficient cyclin D  [ **CCND1** ]  induction in IFN gamma treated cells, resulting in reduced activity of CDK4 and  **CDK2**  kinases and pRB hypophosphorylation.  Ref: 12531694 Exp Cell Res, 2003
- However, the activity of cyclin E  **CDK2**  was decreased in a concentration and time dependent manner and the activity of cyclin D  [ **CCND1** ]  CDK4 was inhibited when serum starved synchronized cells were released from synchronization.  Ref: 12781329 Biochem Pharmacol, 2003
**Neutral relationship**- Upon phosphorylation by G 1 cyclin cyclin dependent kinase Cdk complexes such as cyclin D1  [ **CCND1** ]  Cdk4 6 and cyclin E  **Cdk2** , they lose the ability to bind E2F, and cells are thereby allowed to progress into S phase.  Ref: 12594215 J Biol Chem, 2003
- We further show that Cyclin D  [ **CCND1** ]  Cdk4 as well as Cyclin E  **Cdk2**  binds and regulates STAT92E protein stability.  Ref: 12586062 Dev Cell, 2003
- This cyclin D2 CDK4 complex was also able to bind p27 kip1 in cyclin D1  [ **CCND1** ]  uterine luminal epithelial cells allowing for the activation of  **CDK2** .  Ref: 12649329 Mol Endocrinol< MedlineTA>Mol Endocrinol, 2003
- We propose a mathematical model for the regulation of the G1 phase of the mammalian cell cycle taking into account interactions of cyclin D  [ **CCND1** ]  cdk4, cyclin E  **cdk2** , Rb and E2F.  Ref: 12695688 Cell Cycle< MedlineTA>Cell Cycle, 2003
- The bypass of TNF induced G1 arrest seems to be related to the increase in the stability of cyclin D  [ **CCND1** ]  bound CDK complexes, increasing the total amount of  **CDK2**  4 and 6 complexes and leading to a functional down titration of the p21 waf1 molecules.  Ref: 12761882 J Cell Biochem, 2003

**Non-interactive relationship** (e.g. studied together, co-existance, homology, etc.)

- This effect is accomplished through VES significantly decreasing expression of the cell cycle regulatory proteins cyclin D1  [ **CCND1** ] , D3, and E,  **cdk2**  and 4, but not cdk6.  Ref: 12504091 Biochem Biophys Res Commun, 2003
- Cyclin D  [ **CCND1** ]  Cdk4 and cyclin E  **Cdk2**  regulate the Jak STAT signal transduction pathway in Drosophila.  Ref: 12586062 Dev Cell, 2003
- In the present study, asbestos treated human mesothelial cells HMC, SV40 transformed human mesothelial cells MeT 5A and a human mesothelioma cell line COLO were investigated for genetic changes of cell cycle genes cyclin D1  [ **CCND1** ] , p16INK4a, RB1,  **CDK2**  using multicolor fluorescence in situ hybridization mFISH in interphase cells.  Ref: 12643444 Cell Mol Biol Noisy le grand , 2002
- Western blot analysis revealed that oxLDL stimulated cell proliferation was associated with significant increases in the expression of proteins that regulate entry into and progression through the cell cycle cell division cycle 2, cyclin dependent kinase cdk 2  [ **CDK2** ] , cdk 4, cyclin B1, cyclin D1  [ **CCND1** ] , and PCNA.  Ref: 12529257 Am J Physiol Heart Circ Physiol, 2003
- The preadipocytes traverse the G 1 S checkpoint synchronously as evidenced by the expressionactivation of  **cdk2**  cyclin EA, turnover of p27kip1, hyperphosphorylation of Rb, translocation of cyclin D  [ **CCND1** ]  1 from nuclei to cytoplasm and GSK 3beta from cytoplasm to nuclei, and incorporation of 3 H thymidine into DNA.  Ref: 12502791 Proc Natl Acad Sci U S A, 2003
- In the absence of ligand, a dose dependent increase in p75 NTR protein expression was associated with reduced expression of cyclin D1  [ **CCND1** ] , cyclin E, and cyclin dependent kinase 2  [ **CDK2** ]   **cdk2**  as well as decreased  **cdk2**  activity.  Ref: 12619038 Mol Carcinog, 2003
- RESULTS TNF and IFN gamma synergistically induced a G1 arrest associated with reduced levels of cyclin D1  [ **CCND1** ]  and  **cdk2** , and increased expression of the cdk inhibitors p16INK4a, p21WAF and p27Kip1.  Ref: 12530059 Anticancer Res< MedlineTA>Anticancer Res, 2003
- Bcl  [ **CCND1** ]  2 antiproliferative effect in E1A c Ha ras transformants was not associated with alterations in  **Cdk2** , cyclin E and A contents.  Ref: 12722479 Tsitologiia, 2003
- In contrast, degradation of the AhR in HepG2 liver cancer cells resulted in decreased G0 G1 > S phase progression, and this was accompanied by decreased expression of cyclin D1  [ **CCND1** ] , cyclin E, cyclin dependent kinase 2  [ **CDK2** ]   **cdk2** , and cdk4.  Ref: 12761348 Mol Pharmacol, 2003
- As shown by immunoblot analysis, EGCG treatment of LNCaP and DU145 cells resulted in significant dose and time dependent i upregulation of the protein expression of WAF1 p21, KIP1 p27, INK4a p16, and INK4c p18, ii down modulation of the protein expression of cyclin D1  [ **CCND1** ] , cyclin E,  **cdk2** , cdk4, and cdk6, but not of cyclin D2, iii increase in the binding of cyclin D1  [ **CCND1** ]  toward WAF1 p21 and KIP1 p27, and iv decrease in the binding of cyclin E toward  **cdk2** .  Ref: 12559991 Arch Biochem Biophys, 2003
- Western blot analysis of G 1 phase regulatory proteins demonstrated that the protein levels of cyclin dependent kinase 2  [ **CDK2** ]   **Cdk2** , Cdk4, Cyclin E and Cyclin D3 were decreased after treatment with SCK6 but not those of Cdk6, Cyclin D1  [ **CCND1** ]  and D2.  Ref: 12706452 Eur J Pharmacol, 2003
- In a series of 23 surgical biopsies of grade II and III oligodendroglioma, cyclin D1  [ **CCND1** ] , E, A, B1, CDK4 6,  **CDK2** , Cdc2 and p27 Kip.1 have been studied by immunohistochemistry and Western blot.  Ref: 12873740 Neurosci Lett, 2003
- Overall, our results indicate that Rho kinase dependent stress fiber formation is required for sustained activation of the MEK ERK pathway and the mid G 1 phase induction of cyclin D1  [ **CCND1** ] , but not for other aspects of cdk4 or  **cdk2**  activation.  Ref: 12773570 Mol Cell Biol, 2003
- These data identify c Myc, cyclin D1  [ **CCND1** ] , p21 WAF1 CIP1 and cyclin E  **Cdk2**  as central components of estrogen regulation of cell cycle progression and hence as potential downstream targets that contribute to the role of estrogen in oncogenesis.  Ref: 12790780 Endocr Relat Cancer< MedlineTA>Endocr Relat Cancer, 2003
- Arsenic increased expression of the P21 protein and decreased levels of cyclin A, cyclin B1 and cyclin D1  [ **CCND1** ] , but expression of  **CDK2** , CDK4, CDK6, and cyclin E were not affected.  Ref: 12883267 Pancreas, 2003
- The initiation of this TP53 dependent G1 phase arrest occurs despite the presence of substantial levels of cyclin D1  [ **CCND1** ]  CDK4 and cyclin E  **CDK2**  kinase activities, hyperphosphoryated RB, and active E2F1.  Ref: 12492370 Radiat Res, 2003
- CONCLUSION These results implicate the down regulation of cyclin D1  [ **CCND1** ]  and cdk 2  [ **CDK2** ] , and up regulation of p16INK4a, p21WAF and p27Kip1 in the suppression of endothelial cell proliferation induced by TNF IFN gamma and demonstrate that increased p16INK4a levels are not sufficient to suppress alpha V beta 3 mediated endothelial cell adhesion.  Ref: 12530059 Anticancer Res< MedlineTA>Anticancer Res, 2003
- Binding of these CKI s to both the cyclin D1  [ **CCND1** ]  CDK 4 complex and the cyclin E CDK 2  [ **CDK2** ]  complex were increased and the kinase activities of these complexes were reduced with increasing dietary corticosterone.  Ref: 12807724 Carcinogenesis< MedlineTA>Carcinogenesis, 2003
- The different properties of cells lacking dE2F1 dE2F2 and dE2F1 DAP lead to the surprising observation that dE2F2 mediated repression differs from retinoblastoma family protein 1 RBF1 inhibition of dE2F1, and is resistant to both CycE  **cdk2**  and Cyclin D  [ **CCND1** ]  cyclin dependent kinase 4 CycD cdk4.  Ref: 12651890 Genes Dev, 2003
- Monensin decreased the levels of  **CDK2** , CDK6, cdc2, cyclin A, cyclin B1, cyclin D1  [ **CCND1** ]  and cyclin E proteins but did not alter CDK4 protein.  Ref: 12792794 Int J Oncol, 2003
- Levels of cyclin D1  [ **CCND1** ] , cyclin E, CDK 2  [ **CDK2** ]  and CDK 4 were reduced in carcinomas from corticosterone treated rats.  Ref: 12807724 Carcinogenesis< MedlineTA>Carcinogenesis, 2003
- In the lactacystin treated OLGcs, there was a dose dependent decrease in the number of cells incorporating bromodeoxyuridine and in the activity of the complexes cyclin D  [ **CCND1** ]  cdk4 and cyclin E  **cdk2** .  Ref: 12805303 J Neurosci, 2003
- Positive staining for cyclin D1  [ **CCND1** ] , cyclin E, cyclin A, cyclin B1,  **cdk2** , cdk4, cdc2, p53, p21, and p27 was observed in 63%, 66%, 31%, 32%, 51%, 77%, 71%, 43%, 35%, and 60% of the 82 carcinomas, respectively.  Ref: 12792921 Hum Pathol, 2003
- Although the cyclin D1  [ **CCND1** ]  dependent kinase did not phosphorylate MCM7, active cyclin D1  [ **CCND1** ]  CDK4, but not cyclin E  **CDK2** , did catalyze the dissociation of an RB.MCM7 complex.  Ref: 12519773 J Biol Chem, 2003
- When we examined the effects of this drug on SNU C1 cells, monensin decreased the levels of  **CDK2** , CDK4, CDK6, cyclin D1  [ **CCND1** ]  and cyclin A proteins.  Ref: 12527937 Int J Oncol, 2003
- EGF administration after PH also significantly affected the activity of the cyclin D1  [ **CCND1** ]  cdk4 and cyclin E  **cdk2**  complexes, mainly by changing their time progression it accelerated the increase in activity to 18h and caused a subsequent drop in activity after 34h.  Ref: 12876382 Cell Physiol Biochem, 2003
- Quantitative immunochemical evaluation of the expression of certain proteins controlling the cell cycle showed an increase in p27kip1, cyclin D  [ **CCND1** ] , and cdk4 positive cells, with a decrease in cyclin E and  **cdk2**  positive cells.  Ref: 12805303 J Neurosci, 2003
- These data suggest that Cyclin D  [ **CCND1** ]  Cdk4 and Cyclin E  **Cdk2**  play more versatile roles in Drosophila development.  Ref: 12586062 Dev Cell, 2003
- Modulation of  **cdk2** , cyclin D1  [ **CCND1** ] , p16INK4a, p21WAF and p27Kip1 expression in endothelial cells by TNF IFN gamma.  Ref: 12530059 Anticancer Res< MedlineTA>Anticancer Res, 2003
- Western blotting analysis revealed that the protein levels of cyclin A, cyclin E, and cyclin dependent kinase CDK 2  [ **CDK2** ]  but not cyclin D1  [ **CCND1** ]  and CDK4 decreased after progesterone treatment, but those of CDK inhibitory proteins, p21 and p27, increased.  Ref: 12810531 Endocrinology< MedlineTA>Endocrinology, 2003
- This pathway also mediates GDNF induced decrease in the cyclin dependent kinase inhibitor p27 kip1 expression, resulting in the increased formation of cyclin D1  [ **CCND1** ]  cdk4 and cyclin E  **cdk2**  complexes, followed by hyperphosphorylation of retinoblastoma, a key event for G1 to S phase progression.  Ref: 12595497 J Am Soc Nephrol, 2003
- These results suggest that the suppression of cell cycle progression in the G1 phase by 5GG was mediated in MCF 7 cells, at least in part, by either the inhibition of cyclin D  [ **CCND1** ]  CDK4 and cyclin E  **CDK2**  activity or the induction of the CDK inhibitors p27 Kip and p21 Cip.  Ref: 12781329 Biochem Pharmacol, 2003
- LY CDK inhibitor treated cells also displayed diminished pRb dephosphorylation on  **CDK2**  and CDK4 specific sites, retinoblastoma protein cleavage, and down regulation of cyclin D  [ **CCND1** ] .  Ref: 12702569 Cancer Res, 2003
